# CXCR4 in Cancer and Its Regulation by PPAR*γ*

**DOI:** 10.1155/2008/769413

**Published:** 2008-09-02

**Authors:** Cynthia Lee Richard, Jonathan Blay

**Affiliations:** ^1^Department of Pharmacology, Dalhousie University, Halifax, Nova Scotia, Canada B3H 1X5; ^2^Department of Human Health and Nutritional Sciences, University of Guelph, Guelph, Ontario, Canada N1G 2W1

## Abstract

Chemokines are peptide mediators involved in normal development, hematopoietic and immune regulation, wound healing, and inflammation. Among the chemokines is CXCL12, which binds principally to its receptor CXCR4 and regulates leukocyte precursor homing to bone marrow and other sites. This role of CXCL12/CXCR4 is “commandeered” by cancer cells to facilitate the spread of CXCR4-bearing tumor cells to tissues with high CXCL12 concentrations. High CXCR4 expression by cancer cells predisposes to aggressive spread and metastasis and ultimately to poor patient outcomes. As well as being useful as a marker for disease progression, CXCR4 is a potential target for anticancer therapies. It is possible to interfere directly with the CXCL12:CXCR4 axis using peptide or small-molecular-weight antagonists. A further opportunity is offered by promoting strategies that downregulate CXCR4 pathways: CXCR4 expression in the tumor microenvironment is modulated by factors such as hypoxia, nucleosides, and eicosanoids. Another promising approach is through targeting PPAR to suppress CXCR4 expression. Endogenous PPAR*γ* such as 15-deoxy-Δ^12,14^-PGJ_2_ and synthetic agonists such as the thiazolidinediones both cause downregulation of CXCR4 mRNA and receptor. Adjuvant therapy using PPAR*γ* agonists may, by stimulating PPAR*γ*-dependent downregulation of CXCR4 on cancer cells, slow the rate of metastasis and impact beneficially on disease progression.

## 1. INTRODUCTION

The regulation of the distribution of motile cells in both normal and disease situations depends upon a variety of peptide and nonpeptide mediators, which stimulate cell movement by both directed (chemotaxis) and nondirected (chemokinesis) mechanisms. Amongst these mediators are the chemokines, a class of peptide mediators that play critical roles in normal development, regulation of the hematopoietic and immune systems in the adult, and in repair processes such as wound healing and inflammation. Among the different chemokines is the stromal cell-derived factor-1 (SDF-1), which is now known as CXCL12. CXCL12 binds principally to the receptor CXCR4, although it also acts through the more-recently-described receptor CXCR7 [1]. This review describes the roles of CXCL12 and CXCR4 in normal tissue functions and in cancer, and suggests that the regulation of CXCR4 expression by PPAR*γ* may emerge to be a unique avenue by which a key receptor involved in cancer cell metastasis can be suppressed in a way that will assist with disease therapy.

## 2. CHEMOKINES AND THEIR RECEPTORS IN CELL REGULATION

Chemokines are low-molecular-weight peptide ligands involved in the trafficking of leukocytes and other motile cells [2, 3]. There are four major groups of chemokines, the CXC, CC, C and CX3C chemokines, categorized as such on the basis of their number and spacing of conserved cysteine residues [2, 4]. The nomenclature of chemokines (e.g., “CXCL12") is made up of their subclass (CXC, CC, etc.) followed by “L” for ligand, and a specific number [2, 3].

The receptors for chemokines are cell-surface, seven-transmembrane G protein-coupled receptors [2]. The naming of these receptors (e.g., “CXCR4") is based on the subclass of chemokine that the receptor recognizes, followed by “R” for receptor and a number (which need not correspond to the number assigned to its cognate ligand(s)). There are 19 well-recognized chemokine receptors (e.g., CXCR1-6, CCR1-10, CX_3_CR1, and XCR1) [1, 5]. Many chemokine receptors have more than one known ligand, and many chemokines can activate more than one receptor. Thus, there is much promiscuity in chemokine/receptor signaling.

Chemokines bind within the extracellular domain of the chemokine receptor, which comprises the N-terminus and three extracellular loops [3]. The intracellular domain, which consists of three loops and the C-terminus, associates with G proteins that, upon activation, lead to inhibition of adenylyl cyclase activity [3]. Typical cellular consequences of chemokine binding include changes in gene expression, cell polarization, and chemotaxis (directed cell migration) [4].

Chemokines play a major role in regulating the migration of cells of the immune system, leading to the modulation of immune responses. Their exact role depends on the expression pattern of receptors on specific leukocyte subsets [2] but encompasses the regulation of lymphocyte trafficking, lymphoid tissue development, Th1/Th2 modulation, and the effecting of inflammatory reactions. Chemokine receptors are also found on other cell types, and play a part in stem cell recruitment and angiogenesis, in development and wound healing [4]. When such pathways are subverted in neoplastic cells, chemokines take over prominent roles in the metastatic process, both in terms of the dissemination of cells from primary tumors and in growth of the cancer at metastatic sites. As we will see, this is the case for CXCR4.

## 3. THE CHEMOKINE RECEPTOR CXCR4 AND ITS LIGAND CXCL12 (SDF-1)

The receptor now known as CXCR4 was cloned in 1994, and was originally given the name leukocyte-expressed seven-transmembrane domain receptor (LESTR) due to its abundant expression in several leukocyte populations [6]. It was independently cloned by others and named “fusin” because of its ability to act as a coreceptor for HIV fusion and entry [7]. It further has the designation “CD184” as part of the cluster of differentiation antigens found on activated leukocytes. LESTR/fusin/CD184 was originally considered to be an orphan receptor. However, the chemokine CXCL12, originally termed stromal cell-derived factor 1 (SDF-1), was shown by two independent research groups to be a ligand for LESTR/fusin/CD184, and the name CXCR4 was proposed [8, 9]. The *CXCR4* gene is constitutively expressed, and CXCR4 protein has been detected on many leukocytes, including lymphocytes, monocytes, NK cells, and dendritic cells; as well as on vascular smooth muscle cells, endothelial cells, cells lining the gastrointestinal tract, microglia, neurons, and astrocytes [10–13]. Until recently, CXCR4 was considered to be the only receptor for CXCL12, but the previous orphan receptor RDC1 is now recognized as an additional CXCL12 receptor, for which the name CXCR7 has been given [1]. CXCL12 itself is widely expressed at different levels in many tissues [14].

## 4. CXCL12 AND CXCR4 IN NORMAL TISSUE FUNCTION

The interplay between CXCL12 and CXCR4 is critical to normal development. Indeed (and unlike mice deficient in other chemokine/receptors) mice lacking CXCL12 or CXCR4 die in utero or shortly after birth [2, 15–17]. CXCL12/CXCR4 signaling is required during the development of the hematopoietic, cardiac, vascular, and nervous systems. Absence of this axis in embryonic life leads to defects in bone marrow myeloid cell formation, cardiac dysfunction due to impaired ventricular septum formation, and developmental defects in the cerebellum and in the vasculature of the gastrointestinal tract [15–17].

In the normal adult, CXCL12 and CXCR4 are involved in the homing and retention of hematopoietic progenitor cells in the bone marrow. These progenitor cells express high levels of CXCR4, and are attracted to CXCL12 produced by stromal cells in specialized bone marrow niches [18]. Activating mutations of the *CXCR4* gene lead to aberrant retention of myeloid cells within the bone marrow [19]. CXCL12 also acts as a chemoattractant for stem cells and some differentiated cells in the pathological contexts of inflammation and tissue regeneration/repair [20–24]. It is this function of controlling cell migration and homing that is subverted in cancer.

## 5. CXCL12 AND CXCR4 IN CANCER METASTASIS AND GROWTH

In many ways, the process of metastasis is similar to leukocyte and stem cell trafficking, processes which involve the CXCL12/CXCR4 axis [20]. Cancer cells that express CXCR4 exploit the same signaling pathway, leading to homing and retention in tissues that are rich in CXCL12.

The foundation for our appreciation of the role that CXCR4 and CXCL12 may play in cancer metastasis was set in 2001, when a landmark study by Albert Zlotnik's group demonstrated the importance of the CXCL12/CXCR4 axis in site-specific metastasis of breast cancer [25]. In that study, it was found that CXCR4 expression was low or undetectable in normal epithelial cells, but consistently upregulated in breast cancer cell lines and primary breast cancer cells at both the mRNA and protein level. Human breast carcinoma cells that expressed high levels of CXCR4 underwent morphological changes and migrated directionally in response to CXCL12, indicating that the CXCR4 receptor was active. Crucially, the ligand CXCL12 was highly expressed in tissues taken from human organ sites to which breast cancer cells metastasize, including lymph nodes, lung, liver, and bone marrow, but expressed at low levels in tissues that represent rare sites of metastasis, including the kidney, skin, and muscle. The ability of MDA-MB-231 human breast cancer cells (a cell line that is metastastic in experimental models) to migrate towards protein extracts of lung and liver, or to produce lung and lymph node metastasis after tail-vein injection or orthotopic implantation, was inhibited by neutralizing anti-CXCR4 and/or anti-CXCL12 antibodies. These findings were the first to show the biological importance of this chemokine/receptor pair in the evolution and spread of cancer.

Since that time, the CXCL12/CXCR4 axis has been shown to be important in the progression and spread of more than 25 different cancers. Our present knowledge is based on (i) studies in cellular and animal experimental models, (ii) surveys of human tissues at different stages of cancer progression, and (iii) population-based studies of morbidity and survival. A summary of present data is shown in Table 1.

CXCR4 has been shown to be expressed at high levels on cells of all of the major adult solid epithelial cancers (breast, colorectal, lung, ovary, prostate, etc.). The ability of the cells to colonize other tissues by gaining advantage from CXCR4-dependent mechanisms depends on the presence of CXCL12 in the tissue fluid. Various studies have shown significant CXCL12 concentrations in the fluid-filled cavities through which many cancers disseminate, and at tissue locations in which metastases characteristically develop. Biologically, significant CXCL12 levels have been found in peritoneal ascites from ovarian cancer patients [26], pleural effusions in lung cancer [27], lymph nodes, bone, and lungs as well as other tissues [25, 28, 29].

Detailed studies of the cellular interactions involved in the metastasis of prostate cancer cells to bone [29] have shown that the interaction of CXCL12 with CXCR4 plays a major role in successive steps in the metastatic process. Human osteoblasts express CXCL12 mRNA and protein, whereas prostate cancer cells express CXCR4 mRNA and receptor. Prostate cancer cells that have become disseminated into the circulation respond to the CXCL12-CXCR4 pathway by enhanced adherence to the bone marrow endothelium and migration across endothelial barriers and basement membranes, ultimately adhering to components of the bone marrow in response to a CXCL12 gradient [29]. CXCL12 from osteoblasts has also been shown to act on CXCR4 to induce release of IL-6 from human squamous cell carcinoma cells to promote osteoclastogenesis [30].

As well as promoting the migration of cancer cells and their invasion through physical barriers as well as adherence to target structures, CXCL12 can act upon CXCR4 on the cancer cells to promote cancer cell growth along with other mitogenic factors. This has been shown in cells from colorectal [31], prostate [32], and ovarian [33] cancers. Furthermore, CXCL12 can promote cancer dissemination indirectly by enhancing the vascular supply, since the CXCL12/CXCR4 axis may also promote tumor angiogenesis. Vascular endothelial growth factor (VEGF) and CXCL12 have been shown to increase angiogenesis synergistically in an in vivo Matrigel assay and to promote proliferation and migration of human umbilical vein endothelial cells (HUVECs) in vitro [34].

## 6. THE EFFECT OF CXCL12 ON CELLULAR PROCESSES

Activation of CXCR4 produces specific cellular changes that are consistent with a migratory and invasive cell phenotype. Exposure of cells to CXCL12 produces upregulation of matrix metalloproteinases (MMPs) such as MMP-2 and MMP-9 [25, 26, 29, 35–39]. In addition, CXCL12 enhances adhesion to components of the extracellular matrix such as fibronectin, laminin, and collagen types I/III [37, 40], or to other cell types (e.g., endothelial or bone marrow stromal cells) [29, 41, 42]. These changes are mediated in large part by integrin signaling [29, 43, 44]. Many signaling pathways are activated by CXCL12 downstream of CXCR4 in cancer cells. For example, CXCL12 has been shown to increase ERK1/2 phosphorylation [30, 31, 49, 0.70, 76.78, 79], Akt phosphorylation [50, 77.88], and PI3K activation [45].

## 7. CXCR4 IN BREAST CANCER

CXCR4 is expressed at a low level in normal breast epithelium but becomes more strongly expressed in the early stages of carcinogenesis, showing both a more intense immunohistochemical staining pattern and an altered cellular localization in studies of human ductal carcinoma in situ (DCIS) [46, 47]. An extensive tissue microarray study of 1808 invasive breast carcinomas and 214 pre-invasive breast samples linked to clinical data has shown that the level of CXCR4 expression can be linked to tumor progressivity (tumor grade and lymph node status) and to other prognostic factors such as HER2 expression and hormone receptor (ER and PR) negativity, as well as to patient survival [46]. These observations in human tissues have led to the view that CXCR4 provides a selective advantage to newly formed neoplastic cells in the early primary breast tumor as well as being important to later invasion and metastasis [13, 46–48]. This is consistent with observations in mouse models of breast cancer in which interventions affecting CXCR4 reduced both growth of the primary tumor and metastasis [49].

Prominent CXCR4 expression is a feature of all major histological forms of invasive breast cancer, including ductal, lobular, mucinous [46], and the distinctive and highly aggressive inflammatory form of the disease [50]. Several independent studies have shown that the extent and pattern of CXCR4 expression is related to axillary lymph node involvement in different forms and stages of breast cancer [28, 51–53]. CXCR4 positivity has also been noted as a key feature of breast carcinoma metastasis to bone [54] and brain [55]. The power of CXCR4 as a marker for lymph node metastasis can be greatly increased by concurrently examining the expression of additional markers such as VEGF, MMP-9, and CCR7 [38, 56]. Furthermore, CXCR4 is also one of a subset of markers (the others are uPAR, S100A4, and vimentin) that define highly aggressive and invasive breast carcinoma cells that are associated with malignant pleural or peritoneal effusions in breast cancer patients [57]. CXCR4 expression is therefore a general marker for the spread of breast cancer to its secondary sites, and for aggressive stages of the disease.

There is evidence not only for the use of CXCR4 as a general marker for the progression and metastasis of breast cancer, but also for the identification of individual tumor cells as they are homing from the primary tumor to secondary sites as patients develop metastatic disease. Individual CXCR4-expressing tumor cells have been found in the peripheral blood of breast cancer patients [102], and CXCR4 expression in breast cancer has been associated with the presence of individual tumor cells in the bone marrow of patients [103].

## 8. CXCR4 IN COLORECTAL CANCER

CXCR4 is abundantly expressed by colorectal carcinoma cells [104, 105]. The involvement of CXCR4 expression in colorectal cancer progression was first shown by Roos and colleagues [71]. CT-26 mouse colon carcinoma cells were transfected with CXCL12 extended with a Lys-Asp-Glu-Leu (KDEL) sequence. The KDEL receptor functions to retain resident endoplasmic reticulum (ER) proteins, which contain a C-terminal KDEL sequence, in the ER. With this “intrakine approach," CXCL12-KDEL binds to the KDEL receptor and is retained in the ER, and CXCR4 protein which binds to CXCL12 is also retained in the ER, preventing its expression at the cell-surface [71, 106]. This approach was first developed as a strategy to reduce HIV infection [107]. After intrasplenic injection, CXCL12-KDEL-transfected CT-26 cells, which had reduced cell-surface CXCR4 protein expression, did not form liver metastases, whereas control cells did [71]. The incidence of lung metastasis was also reduced with CXCL12-KDEL-transfected cells, and survival was increased. Interestingly, unlike Zlotnik's group, who had suggested that CXCR4 expression was necessary for the movement of tumor cells to secondary sites [25], Zeelenberg and colleagues found that CXCR4 expression was not required for migration of CT-26 colorectal tumor cells to the lungs, but rather for tumor expansion at secondary sites [71]. Therefore, these authors concluded that CXCR4 is necessary for the outgrowth of colon cancer micrometastases.

Ottaiano and colleagues found that CXCR4 was overexpressed in human colorectal carcinoma tissues compared to normal tissues [40]. Cell-surface CXCR4 protein was also expressed at high levels on SW620, SW48, and SW480 colorectal carcinoma cells, and at moderate levels on Caco-2 and LoVo cells. CXCL12 enhanced the chemotaxis of SW480 cells as well as their adhesion to fibronectin and collagen type I/III, and both effects were blocked with an anti-CXCR4 neutralizing antibody. CXCL12 also induced cytoskeletal changes, proliferation, and ERK1/2 phosphorylation in SW480 cells. Similarly, Schimanski and colleagues found that SW480, SW620, and HT-29 colorectal carcinoma cells expressed CXCR4 protein, as did colorectal carcinoma tissue samples [72]. CXCL12 induced the chemotaxis of SW480 and SW620 cells. Kim and colleagues found that in patients with colorectal cancer with liver metastases, higher CXCR4 expression was found on metastatic tissues compared to the primary tumor [73]. Furthermore, elevated CXCR4 expression in colorectal cancer is associated with disease progression and reduced survival [40, 72, 73, 75].

## 9. THE UTILITY OF CXCR4 AS A MARKER OF TUMOR PROGRESSION

CXCR4 expression has been associated with disease progression, increased recurrence, and reduced survival in many cancer types, as listed in Table 1. As pointed out earlier, CXCR4 protein expression is detectable in the majority of cases of DCIS of the breast, whereas CXCR4 levels are very low in adjacent normal breast epithelium [46]. This suggests that the acquisition of CXCR4 expression may occur very early in malignant transformation, suggesting its potential as a biomarker. As indicated earlier, it has been suggested that CXCR4 expression may be useful as an indicator of prognosis [56, 73].

Although mutations in the *CXCR4* gene have not been reported in the context of cancer, patients with a single nucleotide polymorphism in the 3′ untranslated region of the *CXCL12* gene had reduced incidence of long distance metastasis of epidermoid non-small cell lung cancer (NSCLC) [108].

## 10. PRECLINICAL EFFICACY OF ANTI-CXCR4 TREATMENTS

Several studies have demonstrated the efficacy of strategies designed to reduce CXCR4 expression or inhibit its activity in preclinical models of cancer development and metastasis. A neutralizing anti-CXCR4 antibody prevented metastasis of MDA-MB-231 breast cancer cells in mice [25] and in another study reduced tumor growth after intraperitoneal (IP) injection of Namalwa non-Hodgkin's lymphoma cells [86]. Interestingly, a neutralizing antibody against CXCR4 also inhibited the growth of subcutaneous tumors derived from pancreatic cancer cells that did not themselves express CXCR4, probably because of the ability of the antibody to block CXCR4 on tumor vasculature [109].

CXCR4 peptide antagonists have also proven effective in preclinical cancer models. The CXCR4 peptide antagonist 4F-benzoyl-TN14003 inhibited lung metastasis of MDA-MB-231 breast cancer cells [110], and 4F-benzoyl-TE14011 reduced pulmonary metastasis of B16-BL6 melanoma cells [111]. Murakami and colleagues assessed the contribution of CXCR4 to the metastatic process by transducing B16 murine melanoma cells with CXCR4, followed by IV injection in syngeneic B57BL/6 mice [112]. CXCR4 expression in this context led to increased pulmonary metastasis, which was reduced with the CXCR4 peptide antagonist T22. Liang and colleagues showed that TN14003 itself, which is a 14-mer peptide CXCR4 antagonist, inhibited in vitro invasion of MDA-MB-231 breast cancer cells and lung metastasis after tail vein injection of these cells, without causing any toxicity [113].

Small molecule (nonpeptide) inhibitors of CXCR4 have also been tested in preclinical cancer models. Rubin and colleagues showed that the noncompetitive CXCR4 antagonist AMD3100 inhibited tumor growth after intracranial implantation of Daoy medulloblastoma cells and U87 glioblastoma cells [63] and also inhibited peritoneal carcinomatosis and ascites formation after IP inoculation of NUGC4 human gastric carcinoma cells [78]. In a different approach, blocking the mammalian target of rapamycin (mTOR) pathway downstream of CXCR4 was shown to suppress processes involved in the peritoneal dissemination of gastric cancer [114].

Liang and colleagues also showed the preclinical efficacy of anti-CXCR4 treatments using an RNA-silencing molecular approach [115]. MDA-MB-231 breast cancer cells transfected with siRNA oligonucleotides to knock down CXCR4 were injected into the tail veins of SCID mice. Mice received twice-weekly IV injections of siRNA oligonucleotides to maintain CXCR4 knockdown. The control mice all developed lung metastases, whereas only one of six mice receiving CXCR4 siRNA-transfected cells and followup injections with CXCR4 siRNA developed metastases. Stable knockdown of CXCR4 expression in 4T1 murine breast carcinoma cells using short hairpin RNA reduced orthotopic tumor growth and lung metastasis [49]. Similarly, MDA-MB-231 cells that had undergone stable knockdown of CXCR4 did not form tumors or lung metastases after orthotopic injection into mammary fat pads of SCID mice, whereas CXCR4-positive cells were tumorigenic [116]. NSCLC 95D lung cancer cells in which CXCR4 was knocked down using antisense technology also formed lung metastases in fewer mice after SC injection compared to CXCR4 positive cells [88]. Finally, manipulations of CXCR4 expression have become possible using microRNAs (miRNAs), which are endogenous short RNAs with the ability to repress the translation of target mRNAs [117–119]. The approach of expressing a synthetic miRNA against CXCR4 mRNA to knock down CXCR4 expression has been used successfully in MDA-MB-231 breast cancer cells, HeLa cervical carcinoma cells, and U2-OS osteosarcoma cells [118, 120, 121]. Reduced CXCR4 expression in the breast cancer model was accompanied by reduced migration and invasion of the cells in vitro and fewer lung metastases in vivo [121]. These studies show the importance of CXCR4 expression in both primary and secondary tumor growth.

## 11. CLINICAL ASSESSMENT OF CXCR4-TARGETED REAGENTS

The bicyclam compound AMD3100 was developed as a small molecule CXCR4 antagonist [122]. Although this compound has not yet been fully assessed in clinical trials to determine its therapeutic potential in cancer, it has been examined in small trials in the context of HIV treatment and hematopoietic progenitor cell mobilization [123–128]. One trial with AMD3100 reported one patient with thrombocytopenia, two patients with premature ventricular contractions, and several patients with paresthesias [126]. AMD3100 did not reduce viral load in HIV patients [122], but did effectively increase hematopoietic progenitor cell mobilization [124, 125, 127, 128]. However, the mechanisms of action are under debate and may be unrelated to inhibition of CXCR4 as was first presumed.

## 12. REGULATION OF CXCR4 EXPRESSION BY FACTORS WITHIN THE TUMOR

Zeelenberg and colleagues found that CT-26 murine colon carcinoma cells grown in vitro expressed CXCR4 mRNA, but cell-surface protein levels were not detectable [71]. When the same cells were freshly isolated from lung or liver metastases or from intrasplenic tumors, cell-surface expression was strongly upregulated. This elevated expression was lost after 2–4 days in culture, indicating that it was not due to selection of a subpopulation of cells with a high CXCR4 expression. The authors concluded that CXCR4 expression was induced by the in vivo tumor microenvironment. Although others have shown that metastatic cells maintain high CXCR4 expression when cultured in vitro [129], and indeed CXCR4 has been suggested as a cancer stem cell biomarker [130], as discussed below there is substantial evidence indicating that CXCR4 expression is nevertheless influenced by the tumor microenvironment. Additionally, aberrant activation of signaling pathways within cancer cells, such as those initiated through HER2, can also contribute to elevated CXCR4 expression [131].

Multiple features and factors present in the tumor microenvironment have been shown to regulate CXCR4 expression on tumor cells and other cell types. One such feature is hypoxia [97, 132]. Solid tumors tend to be hypoxic due to structural abnormalities in their vasculature [133]. Staller and colleagues were the first to demonstrate the involvement of hypoxia in the regulation of CXCR4 expression [97]. Their goal was to identify genes regulated by the von Hippel-Lindau tumor suppressor protein (pVHL) in renal cell carcinoma cells. pVHL is often inactivated in renal cell cancer (RCC) leading to constitutive activation of hypoxia-inducible factor-1 (HIF-1) target genes. In a microarray analysis, they found that CXCR4 mRNA expression was suppressed by the reintroduction of functional pVHL into pVHL-deficient A498 RCC cells, an effect that was due to inactivation of HIF-1. CXCR4 protein was also downregulated, resulting in reduced migration of RCC cells towards CXCL12. Hypoxia increased CXCR4 mRNA expression in HEK-293 human embryonic kidney cells and primary human proximal renal tubular epithelial cells, and a hypoxia response element (HRE) was identified within the CXCR4 promoter [97]. The authors speculated that intratumoral hypoxia may lead to increased CXCR4 expression in diverse types of solid tumors, increasing metastasis to distant organs. Shioppa and colleagues found that hypoxia increased CXCR4 mRNA and cell-surface protein expression in several cell types, including monocytes, human monocyte-derived macrophages, tumor-associated macrophages, HUVECs, CAOV3 ovarian carcinoma cells, and MCF-7 breast carcinoma cells, leading to increased migration towards CXCL12 due to the activation of HIF-1 [132].

The hypoxic environment within tumors also leads to high extracellular levels of adenosine (adenine-9-*β*-D-ribofuranoside), a nucleoside that is involved in energy metabolism and comprises the core structure for adenine nucleotides. The concentration of adenosine in the extracellular fluid of solid tumors is about 100-fold that of adjacent normal tissue [134]. Adenosine concentrations in tumors reach levels that can act on any of four subtypes of adenosine-selective, G-protein-coupled receptors: A1, A2a, A2b, and A3 [135]. Adenosine receptors of all four known subtypes are expressed differentially on different cell types within the tumor, including stromal cells, endothelial cells, and infiltrating leukocytes. We have shown that through such receptors, adenosine can have protumor effects directly on cancer cells and also indirectly via other supporting/infiltrating cells [136–139]. Adenosine also acts through A2a and A2b adenosine receptors on human colorectal carcinoma cells to upregulate CXCR4 mRNA expression up to 10-fold, and selectively increase cell-surface CXCR4 protein up to 3-fold [31]. This increase in cell-surface CXCR4 enables the carcinoma cells to migrate toward CXCL12 and enhances their proliferation in response to CXCL12.

One of the further major factors that allows tumor expansion is vascular endothelial growth factor (VEGF), which is also produced in response to hypoxia and which promotes neovascularisation of the tumor. The angiogenic effect of VEGF increases the supply of nutrients and blood-borne growth factors to allow growth of the tumor. There is significant interplay between the roles of VEGF and CXCR4 in tumor expansion. Concomitan high expression of CXCR4 and VEGF has been observed in colorectal [74, 75], breast [38], and ovarian [34] cancers, as well as in glioma [140] and osteosarcoma [91], in each of which it has been linked to increased angiogenesis, invasion, and/or metastasis. Clinical studies have shown that although VEGF and CXCR4 both predispose to lymphatic involvement and nodal metastasis in colorectal cancer, they work through different regulatory strategies [74]. Their collaborative role in angiogenesis parallels a similar joint action in noncancer processes involving neovascularisation (e.g., [141]), and it has been suggested in the context of tumor angiogenesis that their actions may be synergistic [34]. It is not surprising that these two entities are closely linked; VEGF receptors and CXCR4 have common regulatory pathways. For example, interference with Notch signalling leads to downregulation of both VEGF receptor 2 and CXCR4 [142].

The relationship between VEGF and CXCR4 is complex. Firstly, VEGF can promote CXCR4 pathways. VEGF is present in high levels in tumors and may upregulate CXCR4 expression on tumor cells, as has been demonstrated in glioma [143] and breast cancer [144]. In the case of tumor cells, this upregulation of CXCR4 by VEGF can happen through an autocrine mechanism [144]. VEGF can also upregulate CXCR4 on the endothelial cells that may be involved in angiogenesis during tumor expansion [145, 146].

Conversely, the ability of CXCR4 to signal through PI3K/Akt and ERK1/2 provides a route through which VEGF expression may be regulated by CXCR4 [147–149]. Binding of CXCL12 to CXCR4 has been shown to increase cellular secretion of VEGF in ovarian cancer [150], breast cancer [147], prostate cancer [149, 151], and malignant glioma [152]. This phenomenon parallels the ability of the CXCL12/CXCR4 axis to stimulate VEGF secretion in normal lymphohematopoietic cells [153]. One might therefore expect a large part of the antitumor activity of CXCR4 antagonists to be mediated through reduced secretion of VEGF. Indeed, interference with the CXCL12-CXCR4 pathway has been shown to cause downregulation of expression of VEGF [39]. However, blocking the CXCL12/CXCR4 axis in vivo can inhibit tumor growth and angiogenesis without producing alterations in VEGF pathways [109].

Other growth factors whose levels are elevated in tumors may also enhance CXCR4-dependent mechanisms. Tumors have high levels of tumor necrosis factor-*α* (TNF-*α*), derived primarily from tumor-associated macrophages (TAMs) [154–156]. TNF-*α* itself, or macrophages that serve as a source of TNF-*α*, are able to increase CXCR4 mRNA and cell-surface protein expression on ovarian cancer cells [157] and astroglioma cells [158]. A significant correlation between TNF-*α* and CXCR4 expression was found in ovarian cancer biopsies [157]. The increase in CXCR4 at a cellular level appears to be due to TNF-*α*-induced activation of NF-*κ*B signaling and is associated with enhanced migration towards CXCL12 [157]. Therefore, TAMs may contribute to increased CXCR4 expression on cancer cells via production of TNF-*α*.

Finally, polypeptide growth factors that are associated with the extracellular matrix, and indeed components of the extracellular matrix itself, can upregulate CXCR4 on cancer cells. Transforming growth factor-*β* (TGF-*β*) increases cell-surface CXCR4 protein expression on human melanoma cells [35] and we have recently found that FGF-2 upregulates CXCR4 on human colorectal cancer cells (Bseso B and Blay J, manuscript in preparation). Furthermore, type-I collagen and the preparation Matrigel, which is a secreted ECM rich in laminin [159], also increase levels of CXCR4 on melanoma cells [35]. Therefore, interactions with matrix proteins within tumors may also increase CXCR4 expression.

## 13. THE ROLE OF CYCLOOXYGENASE-2 AND PGE_**2**_ IN CANCER 

The shift to malignancy in epithelia and indeed the progression to invasion and metastasis are associated with increased expression of the enzyme cyclooxygenase-2 (COX-2) [160–163]. High COX-2 expression is in cancer is often associated with reduced patient survival [163]. The immediate effect of high COX-2 expression is increased prostaglandin synthesis, particularly prostaglandin E_2_ (PGE_2_) [164], which in experimental models is associated with the production of vascular loops and arches and evidence of abnormal vessel function [165], a phenotype consistent with tumor angiogenesis. Observations of increased expression of angiogenic regulatory genes, including VEGF, ang-1, and ang-2 are consistent with this view [166]. Furthermore, nonsteroidal anti-inflammatory drugs (NSAIDs), which inhibit cyclooxygenases, reduce both tumor incidence and microvessel density in COX-2-expressing mice [166] and reduce cancer progression in preclinical models and clinical trials [167]. Indeed, NSAIDs and COX-2 inhibitors reduce the relative risk of developing colorectal cancer by 40–50% [167–169].

Tumor-promoting effects of COX-2 overexpression appear to be due in large part to increased PGE_2_ production [170–173]. Associated with the increase in COX-2, there is a decreased expression of 15-hydroxyprostaglandin dehydrogenase (15-PGDH), an enzyme involved in the inactivation of PGE_2_, in cancer compared to normal tissues [174], as well as upregulation of cytosolic PLA_2_ (cPLA_2_), which increases the supply of arachidonic acid substrate for COX-2 [175–177]. In addition to promoting angiogenesis, PGE_2_ also stimulates cancer cell proliferation [178, 179], promotes cell migration [180], and causes transactivation of polypeptide growth factor receptors [181].

## 14. OTHER PROSTAGLANDINS IN CANCER

Prostaglandins together with the thromboxanes are classed as prostanoids, and belong to a larger group of compounds referred to as eicosanoids [182]. The main prostanoids apart from PGE_2_ are prostaglandin F_2*α*_ (PGF_2*α*_), prostaglandin D_2_ (PGD_2_), prostaglandin I_2_ (PGI_2_ or prostacyclin), and thromboxane A_2_ (TXA_2_). As well as reflecting changes in COX-2, cPLA_2_, and inactivating enzymes, the levels of different prostanoids in tumors can be modulated by altered expression of specific prostaglandin synthases [183]. Prostaglandins can also be metabolized nonenzymatically to form a range of products both in the body and in cell culture. PGD_2_ can be converted to cyclopentenone J-series prostaglandins, including prostaglandin J_2_ (PGJ_2_), 9-deoxy-Δ^9^,Δ^12‐13,14^-dihydro-PGD_2_ (Δ^12^-PGJ_2_), and 15-deoxy-Δ^12,14^-PGJ_2_ (15dPGJ_2_); PGE_2_ can be converted to prostaglandin A_2_ (PGA_2_) [184–186]. The tumor microenvironment therefore has a rich and varied content of eicosanoid mediators.

## 15. PROSTAGLANDIN EFFECTS ON CANCER CELLS

Although the major focus of attention has been on PGE_2_, a range of eicosanoids acts to restrain tumor growth. Indeed the PGE_2_ metabolite PGA_2_ reduces cell number and induces apoptosis and cell cycle changes in both human breast cancer cells and human epithelial cervical carcinoma cells [187].

**Figure 1:**
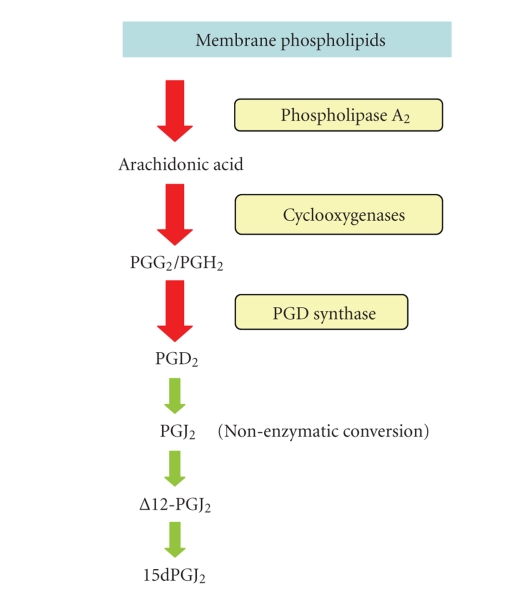
Production of PGD_2_ and conversion to its metabolites. Prostanoids follow an initial common pathway in which arachidonic acid is released from membrane phospholipids by phospholipase A_2_ and then converted to the short-term intermediates PGG_2_ and PGH_2_ by cyclooxygenases. Prostaglandin D synthase forms PGD_2_ itself, but subsequent nonenzymatic reactions in aqueous media lead to the sequential production of prostaglandin J_2_ (PGJ_2_), 9-deoxy-Δ^9^,Δ^12‐13,14^-dihydro-PGD_2_ (Δ^12^‐PGJ_2_), and 15-deoxy-Δ^12,14^‐PGJ_2_ (15dPGJ_2_).

More notably, PGD_2_ and its series of derivatives have anticancer effects. PGD_2_ itself can reduce the growth of carcinoma cells [188]. However, other studies have shown that the nonenzymatic breakdown of PGD_2_ to sequential metabolites (Figure 1) may be required for growth inhibition and that the latter metabolites are the active eicosanoids [189–194]. PGD_2_ therefore can act independently of its DP receptors by its metabolism through a dehydration reaction to prostaglandin J_2_ (PGJ_2_), Δ^12^-PGJ_2_, and then to 15-deoxy-Δ^12,14^-prostaglandin J_2_(15dPGJ_2_) [184]. This reaction occurs in cell culture media, both in the presence and absence of serum [184, 189, 195]. Therefore, it is possible that many effects noted in vitro with PGD_2_ are actually due to the formation of J-series prostaglandins. Frequent replacement with fresh medium containing PGD_2_ in such circumstances can eliminate the response, while the addition of the metabolite(s) themselves leads to growth inhibition in a shorter timeframe than PGD_2_ itself [189]. Some workers have proposed that Δ^12^-PGJ_2_ is the key metabolite [189]; but in fact all of the successive J-series prostaglandins, that is, PGJ_2_, Δ^12^-PGJ_2_, and 15dPGJ_2_, are able to reduce proliferation and induce apoptosis of cancer cells [190]. Furthermore, the end metabolite 15dPGJ_2_ is active against many cell types, including colorectal carcinoma cells [191, 192], prostate carcinoma cells [193], and Burkitt lymphoma cells [194], suggesting that 15dPGJ_2_ may be the crucial mediator.

## 16. THE ROLE OF 15dPGJ_**2**_ AND ITS ACTION ON PPAR*γ*

15dPGJ_2_ is an agonist for the nuclear receptor peroxisome proliferator-activated receptor *γ* (PPAR*γ*) [196, 197], and activation of PPAR*γ* may account for the growth inhibitory effects of 15dPGJ_2_. PPAR*γ* activation results in its heterodimerization with the retinoid X receptor (RXR), binding to peroxisome proliferator response elements (PPREs) on DNA, and subsequent activation of target gene expression [198]. PPAR*γ* is aberrantly expressed in some cancer types [199], and in many cases its activation leads to cell death or differentiation [191, 200, 201]. This action of 15dPGJ_2_, and by extension its precursors PGD_2_, PGJ_2_, and Δ^12^-PGJ_2_, may underlie the major action of these eicosanoids on cell growth. For example, 15dPGJ_2_ reduces the growth of PC-3 human prostate cancer cells through the activation of PPAR*γ* [202]. However, in addition to direct growth-inhibitory effects, 15dPGJ_2_ may also exert anticancer effects by reducing expression of protumor proteins. For example, 15dPGJ_2_ inhibits phorbol ester-induced VEGF and COX-2 expression in SW620 human colorectal carcinoma cells [203].

## 17. 15dPGJ_**2**_ CAUSES DOWNREGULATION OF CXCR4 ON CANCER CELLS

In our studies of the possible effects of these different prostaglandins on CXCR4, we focused upon the expression of the mature protein and furthermore restricted our quantitation exclusively to the receptor that is displayed to the external environment at the cell surface [31]. Cell-surface CXCR4 reflects functional receptor that is coupled to cellular responses [31] rather than the very large intracellular pool of inaccessible receptor protein [72].

**Figure 2:**
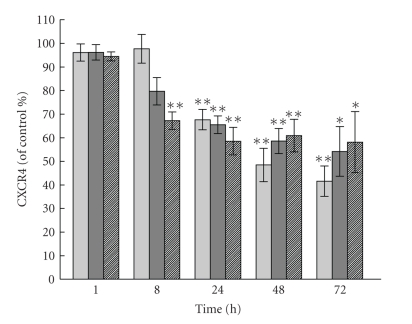
Time course of changes in cell-surface CXCR4 protein expression on HT-29 cells by PGD_2_ and its metabolites. HT-29 cells were treated with vehicle or with 10 *μ*M PGD_2_ (light gray bars), 10 *μ*M PGJ_2_ (dark gray bars), or 3 *μ*M 15dPGJ_2_ (hatched bars), and cell-surface CXCR4 protein expression was measured at the indicated time points. The data shown are expressed relative to the level of CXCR4 receptor on cells treated with vehicle alone at that time point. Values have also been corrected for any possible changes in cell number. The data are mean values ± SE (*n* = 4). Significant decrease due to prostaglandin, **P < .01;*P < .05. The figure is taken from [204] with permission.

Although PGF_2*α*_ (to some extent) and PGE_2_ (as well as its product PGA_2_) have some ability to modulate CXCR4 levels, by far the most potent prostaglandins in this regard are PGD_2_ and its derivatives [204]. Prostaglandin D_2_ and the J-series prostaglandins used at low micromolar concentrations cause substantial loss of CXCR4 from the surface of HT-29 human colorectal carcinoma cells [204]. In particular, 15dPGJ_2_ completely eliminates cell-surface CXCR4 at a concentration of 10^−5^ M in vitro, and has significant effects after a single dose of 300 nM, about 100-fold less than for PGF_2*α*_ [204]. The time course of the decline in cell-surface CXCR4 protein is slow, reaching a maximum only after 48–72 hours (Figure 2). The concentrations of prostaglandins that are needed to cause downregulation after a single dose likely grossly overestimate the steady-state levels that would cause such a response, as we have found in other studies with labile metabolites [31, 138]. We estimate that the effect of 15dPGJ_2_ on CXCR4 is achievable with concentrations of 15dPGJ_2_ present in vivo.

As can be seen in Figure 2, the response to 15dPGJ_2_ occurs more rapidly than that to PGJ_2_, which in turn has a more rapid onset than PGD_2_. We further found that each of these prostaglandins does suppress CXCR4 mRNA expression and that the effect of 15dPGJ_2_ again occurs earlier than that of PGD_2_ [204]. The different relative kinetics of the downregulation of CXCR4 for the J-series prostaglandins are consistent with data on the conversion of PGD_2_ through to 15dPGJ_2_ [189] pointing to 15dPGJ_2_ as the key factor in controlling the levels of functional CXCR4. PGD_2_ produces similar downregulation of CXCR4 in other cell types such as the T47D human breast carcinoma cell line (Richard CL, Blay J, unpublished observations), suggesting that this may be a common phenomenon. The downregulation of CXCR4 expression by 15dPGJ_2_ differs from 15 dPGJ_2_-mediated downregulation of other proteins, including cyclin D1 and estrogen receptor *α*, which has been shown to occur through protein degradation rather than through changes in transcription [205].

## 18. 15dPGJ_**2**_ DOWNREGULATES CXCR4 PRIMARILY VIA PPAR*γ*

The main target for 15dPGJ_2_ is the nuclear receptor PPAR*γ* [196, 197]. We found that the ability of 15dPGJ_2_ to downregulate CXCR4 occurred primarily through this pathway. The effect of 15dPGJ_2_ was mimicked by PPAR*γ* agonists such as rosiglitazone (Table 2, [206]), and antagonized or blocked by the PPAR*γ* antagonists GW9662 and T0070907 [204], which are irreversible inhibitors of PPAR*γ* [207, 208]. A minor part of the downregulatory activity of 15dPGJ_2_ was due to the inhibition of NF*κ*B since the 15dPGJ_2_ analogue CAY10410 (9,10-dihydro-15-deoxy-Δ^12,14^-prostaglandin J_2_) [209, 210], which retains the ability to act on PPAR*γ* but lacks the ability of 15dPGJ_2_ to inhibit NF*κ*B, was less potent than 15dPGJ_2_ [208]. It is the cyclopentenone structure of 15dPGJ_2_ (not present in CAY10410) that confers an ability to inhibit NF*κ*B [211]. Consistent with a role for this structure, cyclopentenone itself (but not cyclopentane or cyclopentene) caused downregulation of CXCR4 [204]. Furthermore, since PGA_2_ possesses the cyclopentenone configuration [212], this explains the ability of PGA_2_ (and that of PGE_2_) to downregulate CXCR4, although it does not contain the *α*,*β*-unsaturated ketone moiety necessary to activate PPAR*γ* signaling [210].

The existence of a mechanism of 15dPGJ_2_-induced CXCR4 downregulation may, in evolutionary terms, be an extension of the anti-inflammatory effects of 15dPGJ_2_. Late in the inflammation process the prostaglandin profile shifts from a PGE_2_-rich state to a PGD_2_-rich (and therefore 15dPGJ_2_-rich) state, leading to the resolution of inflammation [213]. Reduced CXCR4 expression may be an additional mechanism by which 15dPGJ_2_ attempts the resolution of inflammation.

It is clear that this mechanism is not operative in the context of metastatic tumors, because CXCR4 levels are characteristically high (Table 1). Unlike PGE_2_ which is present in elevated concentration in tumors [170–173], 15dPGJ_2_ levels are likely low in tumors compared to normal tissue. Levels of its precursor PGD_2_ are low in tissues of familial adenomatous polyposis, a condition that predisposes to colorectal cancer [172], and have been negatively correlated with hepatic metastasis in tumor tissues taken from patients with colorectal cancer [188]. The enzyme involved in PGD_2_ synthesis, PGD synthase (PGDS), is decreased in cerebrospinal fluid of brain cancer patients compared to patients without disease [214]. There is a contested report of levels of 15dPGJ_2_ being decreased during breast cancer progression, with the lowest levels being detected in metastatic disease [173]. Finally, mechanisms to sequester or eliminate 15dPGJ_2_ may be upregulated in cancer [215, 216]. Overall, it seems that the predominant prostaglandin within tumors is PGE_2_, and 15dPGJ_2_ may not be present in high levels at all. Thus, 15dPGJ_2_-dependent suppression of CXCR4 seems to be a restraint mechanism that is not operative in a cancer situation.

## 19. SYNTHETIC PPAR*γ* AGONISTS DOWNREGULATE CXCR4 ON CANCER CELLS

As indicated above, the PPAR*γ* agonist rosiglitazone also decreased CXCR4 expression on human colorectal cancer cells, congruent with an effect of 15dPGJ_2_ through PPAR*γ*. This effect was seen at both the mRNA and protein level, and was more durable than the effect of 15dPGJ_2_, as it would be expected for a more chemically stable ligand [101, 204]. Moreover, we found that other glitazone agents also downregulate CXCR4, with a rank order of potency (rosiglitazone > pioglitazone > ciglitazone > troglitazone) consistent with their potencies for interaction with PPAR*γ* [206, 217, 218]. Further confirming that these agents were acting through their expected target, PPAR*γ*, and that this target is linked to elimination or reduction of CXCR4 at the cell surface, we showed that the ability of rosiglitazone to decrease CXCR4 was blocked by the PPAR*γ* antagonists GW9662 and T0070907 (Table 2), or by shRNA knockdown of PPAR*γ* expression in the cancer cells [101].

**Figure 3:**
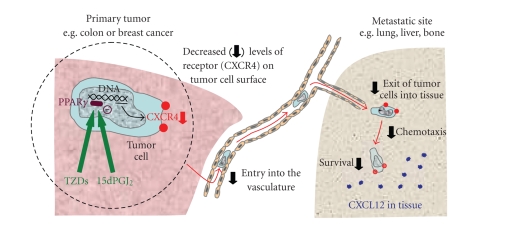
How PPAR*γ* downregulation of CXCR4 may act to decrease metastasis. Tumor cells typically have high levels of CXCR4 at their cell surface. During metastasis, cancer cells that find their way into the bloodstream lodge in tissues that have high concentrations of CXCL12 (e.g., lungs, liver, and bone marrow). CXCL12 both encourages the entry of cells into the tissue and promotes growth of the cell population. Downregulation of CXCR4 by PPAR*γ* activation (endogenous 15dPGJ_2_ or thiazolidinedione drugs, TZDs) will interfere with this process and may impede metastasis.

Therefore, rosiglitazone and its analogues act through PPAR*γ* to cause substantial and persistent suppression of CXCR4 on cancer cells. Since these agents are the same chemicals as the thiazolidinedione (TZD) class of drugs that have been used clinically for the treatment of diabetes (although recent concerns regarding side effects have limited their utility), it opens up the possibility that we may already have a means to manipulate CXCR4 levels in cancer. Given that CXCR4 expression is linked to metastasis, judicious use of TZDs may allow us an opportunity to influence the metastatic process (Figure 3). Recent studies have shown that a unique population of CXCR4+ stem cells may be crucial for expansion of tumor cell populations [130]. We suggest that TZD therapy, by stimulating PPAR*γ*-dependent downregulation of CXCR4 on cancer cells, may slow the rate of metastasis and may impact beneficially on disease progression.

## Figures and Tables

**Table 1:** Involvement of CXCL12/CXCR4 in different cancers.

Cancer	Comments	References
Acute lymphoblastic leukemia	Levels of CXCR4 are elevated on lymphoblasts. Elevated levels of CXCR4 are associated with increased infiltration in liver and spleen	[58]

Acute myelogenous leukemia	High CXCR4 expression is associated with relapse and reduced survival	[59]

Brain cancer	CXCR4 expression is demonstrated in tissues and cell lines derived from glioblastoma, medulloblastoma, and astrocytoma. Cell lines respond to CXCL12 with increased proliferation, survival and migration. Gliomas expressing CXCR4 are associated with increased tumor size and reduced survival	[41, 60–64]

Breast cancer	High CXCR4 expression is noted in breast cancer tissues compared to normal tissues and cell lines with invasive characteristics. CXCR4 expression is associated with more extensive lymph node metastasis and with liver metastasis, although CXCR4 expression in lymph node metastases may be lower than primary cancers. CXCR4 co-expression with HER2/neu is an indicator of more extensive lymph node involvement	[25, 28, 65–67]

Cervical cancer	CXCR4 expression is associated with increased tumor size, stromal invasion, lymph node metastasis, and reduced survival	[68]

Chronic lymphocytic leukemia	Malignant B cells express 3- to 4-fold higher cell-surface CXCR4 levels than normal B cells. High CXCR4 expression on B cells is associated with reduced survival in patients with familial chronic lymphocytic leukemia	[69, 70]

Colorectal cancer	CXCR4 is over-expressed in colorectal carcinoma tissues compared to normal tissues, and on certain established cell lines. In patients with liver metastasis, higher CXCR4 expression is found on liver metastases compared to the primary tumor. In patients with stage I/II disease, high CXCR4 mRNA expression in tumor samples is associated with increased disease recurrence. In patients with stage IV disease, patients with high CXCR4 have decreased overall survival. High CXCR4 expression is associated with increased lymph node involvement and distant metastasis, as well as reduced 3-year survival	[40, 71–75]

Endometrial cancer	Endometrial adenocarcinoma tissues and human cell lines express CXCR4 protein. CXCL12 induces proliferation of endometrial carcinoma cells	[76]

Esophageal cancer	CXCR4 expression is associated with reduced survival and increased lymph node/bone marrow metastasis	[77]

Gastric cancer	A majority of primary gastric tumors and many human gastric carcinoma cell lines express CXCR4. Primary tumors that express CXCR4 protein are associated with peritoneal carcinomatosis	[78]

Head and neck squamous cell cancer	CXCR4 expression is found in tissues and cell lines. High CXCR4 expression is associated with increased occurrence of distant metastases and reduced survival	[79, 80]

Hepatocellular carcinoma	CXCR4 is correlated with tumor progression, metastasis, and reduced survival	[81]

Melanoma	CXCR4 protein is expressed on human melanoma cell lines, as well as on cells isolated from melanoma surgical specimens. CXCL12 enhances cell adhesion to fibronectin, the binding of murine melanoma cells to endothelial cells, and invasion of human melanoma cells across basement membranes. CXCR4 expression is associated with reduced disease-free survival and overall survival	[35, 43, 82, 83]

Multiple myeloma	Multiple myeloma cells isolated from bone marrow and multiple myeloma cell lines express cell-surface CXCR4 protein. CXCL12 enhances adhesion to fibronectin and stimulates cell migration	[84]

Nasopharyngeal cancer	Most primary human nasopharyngeal carcinoma biopsy samples and metastatic lymph nodes stain positively for CXCR4 protein. Nasopharyngeal carcinoma cell lines also express CXCR4 mRNA	[85]

Non-Hodgkin's lymphoma	Most tissue samples and cell lines express high levels of CXCR4 mRNA and cell-surface protein. CXCR4 is implicated in transendothelial migration and proliferation of non-Hodgkin's lymphoma cells	[86]

Nonmelanoma skin cancer	CXCR4 is expressed on invasive squamous cell carcinoma and basal cell carcinoma tissues. Expression on invasive squamous cell carcinoma is increased compared to normal skin	[87]

Non-small cell lung cancer	CXCR4 mRNA is upregulated in NSCLC tissues compared to normal tissues, and levels are higher in tissue samples taken from patients with metastasis than from those without metastasis. Overexpression of CXCR4 in NSCLC cells leads to enhanced migratory, invasive, and adhesive responses to CXCL12. Nuclear CXCR4 staining is associated with longer survival and reduced incidence of metastasis	[88, 89]

Osteosarcoma	CXCR4 mRNA is expressed in most human osteosarcoma samples, and two of three osteosarcoma cell lines. CXCR4 expression is higher at metastatic sites than in the primary tumor	[90, 91]

Ovarian cancer	CXCR4 mRNA is expressed in ovarian cancer cell lines, as well as in biopsies from primary tumors and ovarian cancer ascites. High levels of CXCL12 are present in ascitic fluid taken from patients with ovarian cancer. CXCL12 stimulates the growth of ovarian cancer cells. CXCR4 expression is associated with increased recurrence and reduced survival	[26, 33, 92]

Pancreatic cancer	Most human pancreatic cancer tissues stain positively for CXCR4 expression, and more than half of pancreatic cancer cell lines express CXCR4 mRNA and cell-surface protein. CXCL12 induces chemotaxis of human pancreatic carcinoma cells, as well as stimulates proliferation and promoted survival	[42, 93]

Prostate cancer	Prostate cancer cell lines express CXCR4 mRNA and protein, and approximately half of prostate cancer tissues stain positively for CXCR4. Treatment of cells with CXCL12 increases their adherence to osteosarcoma cells and bone marrow endothelial cells, transendothelial migration, and invasion into Matrigel. CXCR4 expression is a positive predictor of bone metastasis, particularly in patients with elevated prostate specific antigen (PSA) levels. High CXCR4 expression is associated with increased cancer-specific mortality	[29, 36, 94, 95]

Renal cell cancer	One of four human renal cell cancer lines express CXCR4 mRNA, which is upregulated in renal cell cancer tumor samples compared to normal tissue. High CXCR4 expression is associated with poor tumor-specific survival, independent of tumour stage and differentiation grade	[96, 97]

Rhabdomyo sarcoma	Several rhabdomyosarcoma cell lines express cell-surface CXCR4 protein. CXCL12 increases cell motility, induces chemotaxis, increases adhesion to extracellular matrix, and stimulates secretion of MMP-2	[37]

Small cell lung cancer	CXCR4 mRNA and cell-surface protein are detected in cell lines. CXCL12 induces proliferation, increases adherence and motility, and induces morphological changes such as filopodia formation	[98]

Thyroid cancer	Human thyroid carcinoma cell lines express CXCR4 protein, and CXCR4 is upregulated in primary papillary thyroid carcinomas compared to normal thyroid tissue. CXCL12 increases proliferation, inhibits apoptosis, and increases migration and invasion of human thyroid cancer cells	[99, 100]

**Table 2:** Rosiglitazone downregulation of CXCR4 on HT-29 cells and suppression by PPAR*γ* antagonists. HT-29 cells were treated with the PPAR*γ* antagonists (I) GW9662 at 1 *μ*M or (II) T0070907 at 100 nM for 30 minutes before exposure to rosiglitazone (10 nM). Cell-surface CXCR4 protein expression was measured after 48 hours. The data are mean values ± SE (*n* = 4). The table is taken from [101] with permission.

Experiment	PPAR*γ* antagonist	Treatment		Decrease due to rosiglitazone (%)
		Control	Rosiglitazone	
I	Control	2.53 ± 0.14	0.95 ± 0.09***	63
	GW9662	2.47 ± 0.22	2.43 ± 0.27 n.s.	2

II	Control	1.90 ± 0.17	0.81 ± 0.11**	57
	T0070907	2.74 ± 0.17	3.07 ± 0.18 n.s.	0

Significant change due to rosiglitazone, ***P < .001; **P < .01; n.s.: not significant.

## ABBREVIATIONS

CAY10410: 9,10-dihydro-15-deoxy-Δ^12,14^-prostaglandin J_2_
CXCL12: CXC chemokine ligand 12
CXCR4: CXC chemokine receptor 4
DCIS: Ductal carcinoma in situ
GW9662: 2-chloro-5-nitro-N-phenylbenzamide
HIF-1: Hypoxia-inducible factor-1
HRE: Hypoxia response element
LESTR: Leukocyte-expressed seven-transmembrane domain receptor
NF-*κ*B: Nuclear factor-*κ*B
NSAIDs: Nonsteroidal anti-inflammatory drugs
NSCLC: Non-small cell lung cancer
PPAR*γ*: Peroxisome proliferator-activated receptor *γ*
PPRE: Peroxisome proliferator response element
pVHL: Von Hippel-Lindau tumor suppressor protein
RCC: Renal cell cancer
RXR: Retinoid X receptor
SDF-1: Stromal cell-derived factor 1
T0070907: 2-chloro-5-nitro-N-(4-pyridyl)benzamide
TAM: Tumor-associated macrophages
VEGF: Vascular endothelial growth factor
15dPGJ_2_: 15-deoxy-Δ^12,14^‐PGJ_2_
15-PGDH: 15-hydroxyprostaglandin dehydrogenase
Δ^12^‐PGJ_2_: 9-deoxy-Δ^9^,Δ^12‐13,14^-dihydro-PGD_2_.
